# Clinically relevant sequence types of carbapenemase-producing *Escherichia coli* and *Klebsiella pneumoniae* detected in Finnish wastewater in 2021–2022

**DOI:** 10.1186/s13756-024-01370-z

**Published:** 2024-01-30

**Authors:** Viivi Heljanko, Olga Tyni, Venla Johansson, Jussa-Pekka Virtanen, Kati Räisänen, Kirsi-Maarit Lehto, Anssi Lipponen, Sami Oikarinen, Tarja Pitkänen, WastPan Study Group, Ahmad Al-Mustapha, Paula Kurittu, Annika Länsivaara, Rafiqul Hyder, Erja Janhonen, Ananda Tiwari, Anna-Maria Hokajärvi, Aleksi Kolehmainen, Teemu Möttönen, Oskari Luomala, Aapo Juutinen, Soile Blomqvist, Carita Savolainen-Kopra, Anniina Sarekoski, Annamari Heikinheimo

**Affiliations:** 1 Department of Food Hygiene and Environmental Health, Faculty of Veterinary Medicine, University of Helsinki, Helsinki, Finland; 2 Finnish Food Authority, Seinäjoki, Finland; 3 Department of Health Security, Finnish Institute for Health and Welfare, Helsinki, Finland; 4 Faculty of Medicine and Health Technology, Tampere University, Tampere, Finland; 5 Department of Health Security, Finnish Institute for Health and Welfare, Kuopio, Finland

**Keywords:** Wastewater surveillance, Antimicrobial resistance, Carbapenemase-producing *Escherichia coli*, Carbapenemase-producing *Klebsiella pneumoniae*, Carbapenemase-producing Gram-negative bacteria

## Abstract

**Background:**

Antimicrobial resistance (AMR) is a critical threat to human health. *Escherichia coli* and *Klebsiella pneumoniae* are clinically the most important species associated with AMR and are the most common carbapenemase-producing (CP) Enterobacterales detected in human specimens in Finland. Wastewater surveillance has emerged as a potential approach for population-level surveillance of AMR, as wastewater could offer a reflection from a larger population with one sample and minimal recognized ethical issues. In this study, we investigated the potential of wastewater surveillance to detect CP *E. coli* and *K. pneumoniae* strains similar to those detected in human specimens.

**Methods:**

Altogether, 89 composite samples of untreated community wastewater were collected from 10 wastewater treatment plants across Finland in 2021–2022. CP *E. coli* and *K. pneumoniae* were isolated using selective culture media and identified using MALDI-TOF MS. Antimicrobial susceptibility testing was performed using disk diffusion test and broth microdilution method, and a subset of isolates was characterized using whole-genome sequencing.

**Results:**

CP *E. coli* was detected in 26 (29.2%) and *K. pneumoniae* in 25 (28.1%) samples. Among *E. coli,* the most common sequence type (ST) was ST410 (*n* = 7/26, 26.9%), while ST359 (*n* = 4/25, 16.0%) predominated among *K. pneumoniae*. Globally successful STs were detected in both *E. coli* (ST410, ST1284, ST167, and ST405) and *K. pneumoniae* (ST512, ST101, and ST307). *K. pneumoniae* carbapenemases (KPC) were the most common carbapenemases in both *E. coli* (*n* = 11/26, 42.3%) and *K. pneumoniae* (*n* = 13/25, 52.0%), yet also other carbapenemases, such as *bla*_NDM-5,_ *bla*_OXA-48,_ and *bla*_OXA-181_, were detected. We detected isolates harboring similar ST and enzyme type combinations previously linked to clusters in Finland, such as *E. coli* ST410 with *bla*_KPC-2_ and *K. pneumoniae* ST512 with *bla*_KPC-3_.

**Conclusions:**

Our study highlights the presence of clinically relevant strains of CP *E. coli* and *K. pneumoniae* in community wastewater. The results indicate that wastewater surveillance could serve as a monitoring tool for CP Enterobacterales. However, the specificity and sensitivity of the methods should be improved, and technologies, like advanced sequencing methods, should be utilized to distinguish data with public health relevance, harness the full potential of wastewater surveillance, and implement the data in public health surveillance.

**Supplementary Information:**

The online version contains supplementary material available at 10.1186/s13756-024-01370-z.

## Background

Antimicrobial resistance (AMR) is a significant threat to global health [1]. In 2019 alone, AMR was estimated to be responsible for approximately 4.95 million deaths [2]. *Escherichia coli* and *Klebsiella pneumoniae*, among the leading pathogens associated with AMR, are of particular concern. These species belonged to the top five bacterial pathogens responsible for infection-related deaths in 2019, each responsible for over 500 000 deaths globally [2, 3]. They both rank also in the top three bacterial species causing the largest burden of disease estimated by the European Centre for Disease Prevention and Control (ECDC) [4]. *E. coli* and *K. pneumoniae* are Gram-negative opportunistic pathogens belonging to Enterobacterales and are part of the normal microbiota in human and animal gastrointestinal tracts [5, 6]. Additionally, they are found in fecally contaminated environmental sources like soil and water [6, 7].

Carbapenem resistance, emerging in Enterobacterales [8], is a critical threat to human health [9]. Generally, the resistance percentage in all bacterial species in Finland is low (in 2021: 6.4%) [10], and none of the *E. coli* or *K. pneumoniae* isolated from blood was resistant to meropenem in 2021 [11]. However, the number of detected carbapenem-resistant isolates from different sample types has increased in recent years [12], and the emergence of carbapenem resistance is a worrisome threat also in Finland. As in the global situation, *E. coli* and *K. pneumoniae* are the most common carbapenemase-producing Enterobacterales (CPE) in Finland, comprising 48% and 45% of CPE isolates in human specimens in 2022, respectively [12]. Certain sequence types (ST) of *E. coli* and *K. pneumoniae*, known as globally dominant STs, have considerable clinical relevance, i.e., are more frequently detected in clinical samples. Globally dominant STs of Carbapenemase-producing (CP) *E. coli* (e.g., ST410, ST131, ST1284, ST167, and ST405) and CP *K. pneumoniae* (e.g., ST512, ST437, ST258, ST11, ST15, ST101, ST307, and ST147) [13–15] have been detected in human specimens in Finland [12, 16, 17].

The dissemination of carbapenemase-encoding genes is a major concern, as they have the potential to rapidly spread within and between bacterial species due to their frequent location on plasmids [18]. *K. pneumoniae* carbapenemases (KPC) are particularly successful in this regard [19]. In Finland, KPC are the most common carbapenemases found in human specimens [12], with *bla*_KPC-3_ being the most prevalent type in 2012–2018 [16]. However, plasmid-mediated New Delhi metallo-β-lactamases (NDM) and oxacillinase-48-type carbapenemases (OXA-48-like) are also widespread and common in Northern Europe, including Finland [12, 20].

The ongoing but evolving nature of AMR and the lack of clear epidemic peaks, as detected in viral pandemics, often lead to its oversight. Hence, AMR is commonly referred to as the silent pandemic [21]. Current AMR surveillance primarily focuses on bacteria that cause healthcare-associated infections and aims to detect the potential threats these bacteria could create to the population. Consequently, this could result in poorly monitored and understood AMR prevalence in the healthy population [22], and relying only on current AMR surveillance data could lead to potential biases from the population surveillance perspective [23]. To address this gap, alternative options for AMR surveillance at the population level, such as wastewater surveillance (WWS), have been explored [23]. WWS offers the potential for population-level assessment and survey of AMR while avoiding the ethical issues associated with sampling of individuals [24]. However, it is important to understand that AMR and AMR-related genes, both intrinsic and acquired, occur in multiple bacterial species, including clinically less relevant species that are abundant in wastewater [25].

**Fig. 1:**
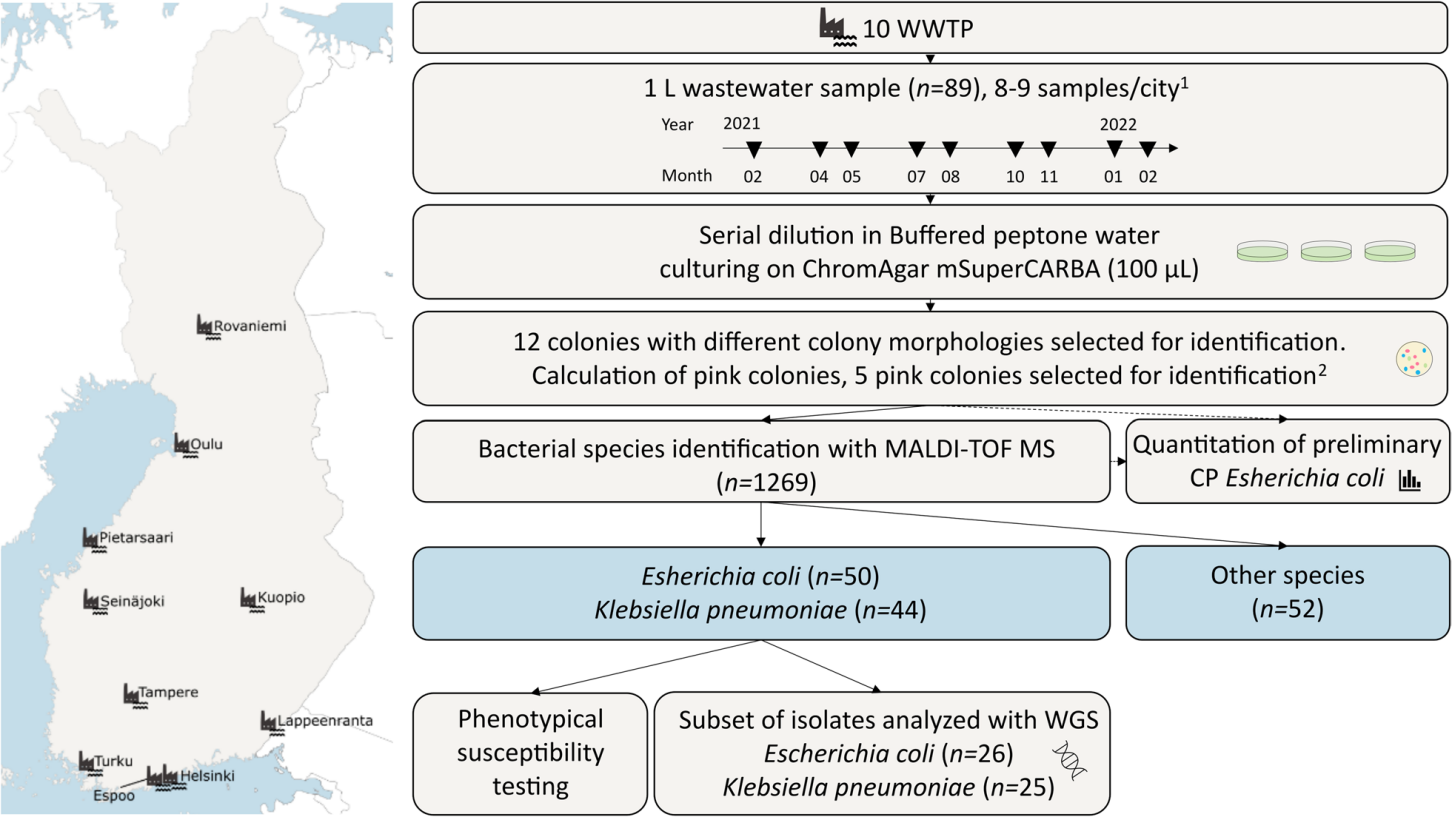
Illustration of the locations of the included wastewater treatment plants (WWTP), the timeline of the sample collection, and the workflow of the study. CP, carbapenemase-producing. WGS, whole-genome sequencing. ^1^One sample missing.^2^If applicable

In this study, we focused on evaluating the occurrence and characteristics of carbapenemase-producing (CP) *E. coli* and *K. pneumoniae* in Finnish community wastewater influent. There has been an increasing recognition of various CP organisms globally. However, CP *E. coli* and *K. pneumoniae* are the most prevalent CP organisms in clinical samples in Finland, having a high clinical relevance. With a combination of culture-based and molecular methods, we investigated the phenotypical and genotypical features of CP *E. coli* and *K. pneumoniae* in 89 wastewater samples collected before any treatment from ten wastewater treatment plants (WWTP) across Finland during 2021–2022 (Fig. 1).

## Methods

### Sample collection, bacterial isolation, and quantitation of preliminary CP *E. coli*

A total of 89 samples of community wastewater influent were collected from 10 different WWTP across Finland between February 2021 and February 2022. The included WWTP serve around 40% of the Finnish population and are distributed across the country [26]. Among these samples, 86 were 24-h composite samples, while, due to sporadic issues with composite collectors, one was a 4-h and one 6-h composite sample, and one was a grab sample (Table 1). The wastewater collection was conducted as part of the “WastPan” consortium project [26]. A 1 L sample was delivered to the laboratory in a cold container with ice packs and processed within 24 h of collection.

**Table 1:** Carbapenemase-producing *Escherichia coli* and *Klebsiella pneumoniae* recovered from wastewater samples across Finland

	Wastewater samples	*Escherichia coli*			*Klebsiella pneumoniae*		
		Positive samples		Isolates	Positive samples		Isolates
City	*n*	*n*	%	*n*	*n*	%	*n*
Espoo	9	1	11.1	1	2	22.2	5
Helsinki	9	6	66.7	12	4	44.4	6
Kuopio	8*	3	37.5	3	1	37.5	1
Lappeenranta	9	–	–	–	2	22.2	2
Oulu	9	2	22.2	2	2	22.2	3
Pietarsaari	9*	3	33.3	4	–	–	–
Rovaniemi	9*	–	–	–	7	77.8	19
Seinäjoki	9	1	11.1	5	–	–	–
Tampere	9*	7	77.8	20	3	33.3	4
Turku	9	3	33.3	3	4	44.4	4
Total	89	26	29.2	50	25	28.1	44

Number (n) and proportion (%). Asterisk (*) indicating deviations in the sampling; Kuopio: One sample missing (October 2021); Pietarsaari: One grab sample (February 2022); Rovaniemi: One 4-h composite sample (August 2021); Tampere: One 6-h composite sample (August 2021)

In the laboratory, a serial dilution in buffered peptone water (BPW) (Oxoid, Basingstoke, Hampshire, United Kingdom) was prepared using dilutions of 10^–1^, 10^–2^, and in summer months also 10^–3^. An aliquot of 100 µL from the undiluted sample and each dilution was plated on individual CHROMagar mSuperCARBA (CHROMagar, Paris, France) plates and incubated aerobically for 18–24 h at 37 °C. The colony morphology was observed following the manufacturer’s instructions, and up to 12 colonies showing different colony morphology were selected. The main objective was to identify *E. coli* and *K. pneumoniae,* typically showing dark pink to reddish and blue colonies, respectively. An additional objective was to identify other bacteria belonging to ESKAPE-E [27], including *Acinetobacter baumannii, Pseudomonas aeruginosa,* and *Enterobacter cloacae,* showing typically cream, translucent, and blue colonies, respectively. The colonies were subcultured with a 1 µL sterile loop on CHROMagar mSuperCARBA and incubated aerobically for 18–24 h at 37 °C until a pure culture was obtained. Lastly, isolates were subcultured on bovine blood agar plates (Columbia Blood Agar Base, Oxoid Ltd., Basingstoke, United Kingdom) and incubated aerobically for 18–24 h at 37 °C for further characterization.

The quantitation of preliminary CP *E. coli* was conducted by counting the colonies showing the characteristic appearance of *E. coli* (dark pink to reddish) on plates containing 10–100 typical colonies. From each sample, five typical colonies were selected and subcultured to achieve pure cultures, following the procedure described above (Fig. 1).

### Bacterial species identification

Isolates were identified with a matrix-assisted laser desorption/ionization time-of-flight mass spectrometry (MALDI-TOF MS) -based Bruker Microflex LT/SH (Bruker Daltonics GmbH & Co. KG, Bremen, Germany). A score value of > 2.0 was considered high confidence, as per the manufacturer’s instructions, and set as the criterion.

### Antimicrobial susceptibility testing

Susceptibility to carbapenems was tested for all presumptive CP *E. coli* and *K. pneumoniae* isolates with meropenem (10 µg) (Abtek Biologicals Ltd, Liverpool, United Kingdom) and ertapenem (10 µg) (Oxoid, Basingstoke, United Kingdom) with a disk diffusion test according to the EUCAST (European Committee of Antimicrobial Susceptibility Testing) standard [28]. Furthermore, antimicrobial susceptibility testing was performed with the broth microdilution method using Sensititre EURGNCOL plates (Thermo Fischer Scientific, East Grinstead, United Kingdom) to determine the minimum inhibitory concentration (MIC) of colistin, piperacillin/tazobactam, ceftazidime/avibactam, ceftolozane/tazobactam, and meropenem. The method was performed according to the manufacturer’s instructions, except for using 0.9% saline instead of sterile water. *E. coli* ATCC 25922 was included as a quality control for each patch of Müeller-Hinton agars. The results were interpreted according to EUCAST epidemiological cut-off values (ECOFFs) [29].

### DNA extraction and whole-genome sequencing (WGS)

In total, 26 *E. coli* and 25 K*. pneumoniae* isolates were subjected to whole-genome sequencing (WGS). One isolate of each species from each city on each sampling month was chosen, if applicable. If multiple isolates were detected, the selection criteria were as follows: (1) isolate with the highest MIC value for meropenem, (2) isolate with the smallest inhibition zone for meropenem, and (3) isolate that was first selected from the primary agar plate. Strains were grown in Tryptone Soya Broth (Oxoid, Basingstoke, United Kingdom) at 37 °C for 16 h and the DNA was extracted from cells harvested from 1 mL of culture by using QIAcube Connect instrument (QIAGEN, Hilden, Germany) with DNeasy Blood & Tissue kit (QIAGEN, Valencia, CA, USA). The quality of DNA was assessed by using a NanoDrop ND-1000 spectrophotometer (Thermo Fischer Scientific, Wilmington, DE, USA) based on a 260/280 ratio. DNA quantity was measured using a Qubit 2.0 fluorometer (Invitrogen, Life Technologies, Carlsbad, CA, USA). Library preparation was performed with a NEBNext Ultra DNA Library Prep Kit for Illumina with 300 bp fragment length. Sequencing was performed with Illumina NovaSeq 6000 (outsourced to Novogene, Cambridge, United Kingdom) with targeted genomic coverage of 100 × and 2 × 150 bp read length.

### Bioinformatical analyses

All (*n* = 51) sequenced isolates were analyzed with Ridom SeqSphere + software v7.7.5 (Ridom GmbH, Germany) [30]. Quality analysis of the sequences was performed with FastQC v0.1.1.7 [31] and adapters were removed with Trimmomatic v0.36 [32]. Raw reads were assembled with SKESA v2.3.0 using default settings [33], and quality trimming was performed with an average quality of ≥ 30 and a window of 20 bases. Remapping and polishing were performed with the BWA-MEM mapping algorithm. Sequencing statistics are presented in Additional file 1. Acquired AMR genes were identified from assembled genomes with NCBI AMRFinderPlus 3.2.3 [34], using 100% alignment and > 90% identity. STs were analyzed by using multilocus sequence types (MLST) [35] in Ridom SeqSphere + (Ridom, Munster, Germany). Warwick MLST scheme was chosen for *E. coli* isolates. *E. coli* isolates with novel STs were submitted to Enterobase [36] and *K. pneumoniae* isolates to Institut Pasteur [37, 38] to assign new STs. Phylogenetic analysis was conducted for all *E. coli* and *K. pneumoniae* isolates with core genome multilocus sequence typing (cgMLST) by comparing 2513 and 2365 alleles with pairwise missing values, respectively. A cluster threshold was determined by 10 allelic differences [39].

## Results

### Detection of *E. coli* and K. pneumoniae with reduced susceptibility to carbapenems

In total, 50 *E. coli* isolates from 26 wastewater samples (*n* = 26/89, 29.2%) and 44 K*. pneumoniae* isolates from 25 wastewater samples (*n* = 25/89, 28.1%) were recovered from CHROMagar mSuperCARBA during the study period (Table 1). Up to five *E. coli* and four *K. pneumoniae* isolates were recovered in each sample. An additional 52 species were identified, including *A. baumannii* and *E. cloacae,* but not *P. aeruginosa* (Additional file 2).

Quantity of preliminary CP *E. coli* was under the detection limit in 62 samples (*n* = 62/89, 69.7%) and peaked at 6.0 × 10^2^ colony forming units/mL (Additional file 3).

### Antimicrobial susceptibility

Disk diffusion test and broth microdilution were performed for all *E. coli* and *K. pneumoniae* isolates. In total, 44 *E. coli* (*n* = 44/50, 88.0%) and 43 K*. pneumoniae* (*n* = 43/44, 97.7%) isolates were considered resistant against meropenem according to the broth microdilution method. Phenotypical resistance against colistin was expressed by four *E. coli* (*n* = 4/50, 8.0%) and eleven *K. pneumoniae* (*n* = 11/44, 25.0%) isolates. Distributions of MICs are presented in Table 2. MIC values and inhibition zones for individual isolates are presented in Additional file 4.

**Table 2:**
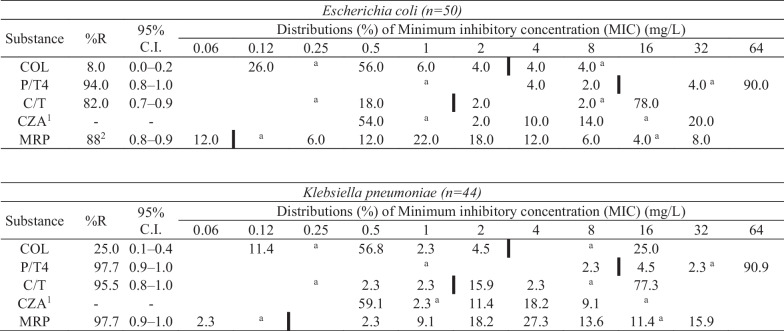
Distribution of MICs for *Escherichia coli* and *Klebsiella pneumoniae* recovered from wastewater.

Bold vertical lines indicate epidemiological cut-off values (ECOFF) (20.7.2023) for resistance for *Escherichia coli* and *Klebsiella pneumoniae*. ^a^ indicating the dilution range tested for each substance. Values below or above the range (^a^) denote Minimum inhibitory concentration (MIC) values smaller or greater than the lowest and highest concentration in the range. ^1^ECOFF value not provided. ^2^Tentative %R; ECOFF value lower (0.06) than the lowest concentration of the test (0.12). %R, proportion of resistant isolates. C.I., confidence interval. COL, colistin. P/T4, Piperacillin/tazobactam constant 4. C/T, Ceftolozane/tazobactam 4. CZA, Ceftazidime/avibactam. MRP, Meropenem

### Multilocus sequence types, antimicrobial resistance genes, and phylogenetics

**Fig. 2:**
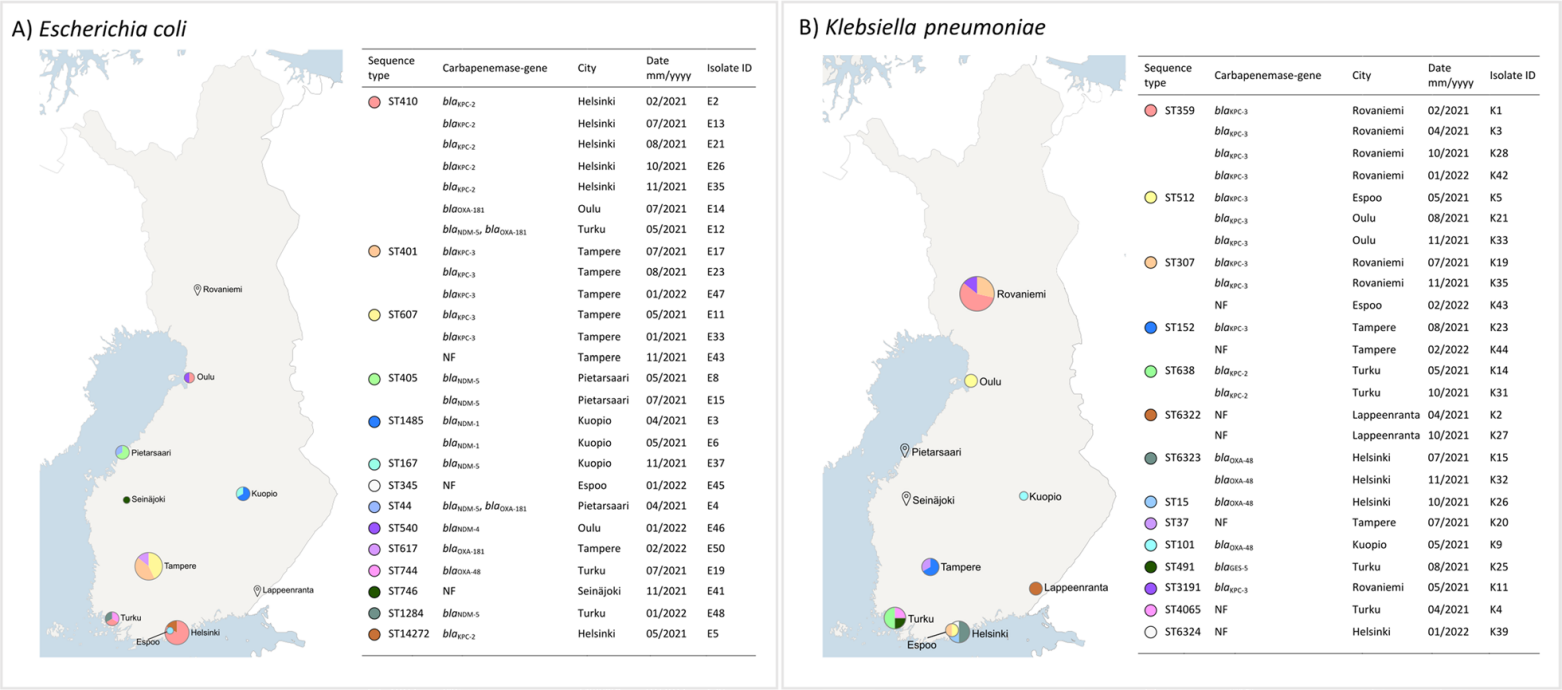
Distribution of sequence types and carbapenemase genes in carbapenemase-producing (CP) *Escherichia coli* (*n* = 26) and *Klebsiella pneumoniae* (*n* = 25) isolates. Geographical distribution of sequence types (ST) across the studied wastewater treatment plants (WWTP), distribution of carbapenemase genes in different STs and detection time of Carbapenemase-producing (**A**) *Escherichia coli* and (**B**) *Klebsiella pneumoniae.* The size of the circle reflects the number of isolates. Location tick marks on the map indicate that no isolates were detected in the corresponding WWTP. Isolate identification numbers (ID) are indicated (e.g., E2). ST, sequence type

In total, 14 different STs of *E. coli* and 14 of *K. pneumoniae* were identified. In *E. coli,* the most prevalent was ST410 (*n* = 7/26, 26.9%), followed by ST401 (*n* = 3/26, 11.5%) and ST607 (*n* = 3/26, 11.5%). In *K. pneumoniae,* the most prevalent was ST359 (*n* = 4/25, 16.0%), followed by ST512 (*n* = 3/25, 12.0%) and ST307 (*n* = 3/25, 12.0%) (Fig. 2A and 2B).

In total, 23 *E. coli* (*n* = 23/26, 88.5%) and 18 K*. pneumoniae* (*n* = 18, 72.0%) isolates were confirmed to carry carbapenemase-encoding genes. In *E. coli,* the most prevalent was *bla*_KPC-2_ (*n* = 6/26, 23.1%), followed by *bla*_KPC-3_ (*n* = 5/26, 19.2%) and *bla*_NDM-5_ (*n* = 4/26, 17.4%). In *K. pneumoniae,* the most prevalent was *bla*_KPC-3_ (*n* = 11/25, 44.0%), followed by *bla*_OXA-48_ (*n* = 4/25, 16.0%) (Fig. 2A and 2B).

**Fig. 3:**
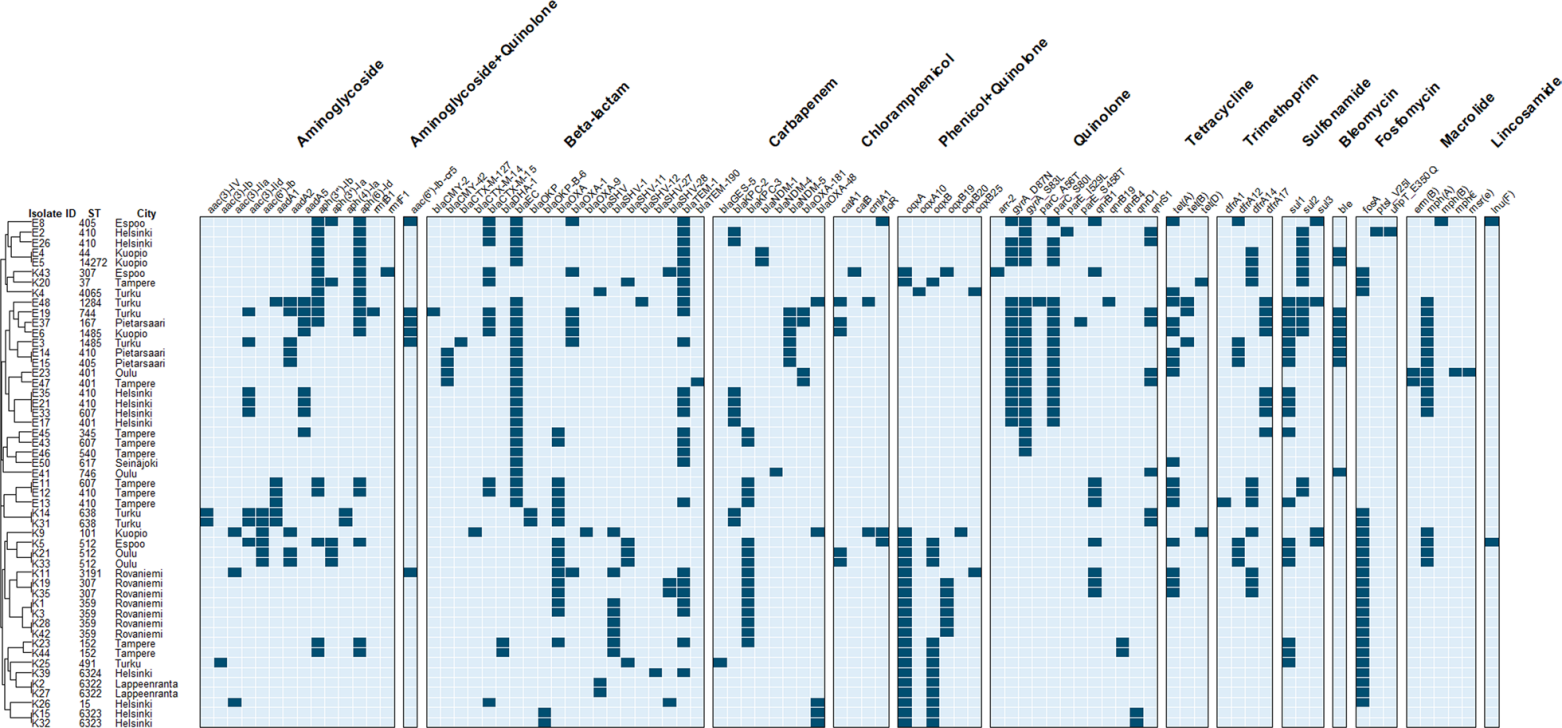
Heatmap of the presence of antimicrobial resistance genes (dark blue) in whole-genome sequenced isolates of carbapenemase-producing *Escherichia coli* (*n* = 26, indicated with Isolate IDs E2–E50) and *Klebsiella pneumoniae* isolates (*n* = 25, indicated with Isolate IDs K1–K44) from wastewater treatment plants across Finland (*n* = 10). The dendrogram is based on the similarity of resistance gene profiles between the isolates

All isolates carried at least one additional beta-lactamase gene, including Extended-Spectrum Beta-Lactamases such as *bla*_CTX-M-15,_ *bla*_CTX-M-14_, and *bla*_SHV-27_ (Fig. 3). All *K. pneumoniae* isolates and 22 (84.6%) of the *E. coli* isolates were multidrug resistant (i.e., harbored resistance genes to at least one agent in three or more antimicrobial categories [40]). Known genes related to colistin resistance (mcr) were not found.

CgMLST revealed closely related strains (< 10 allele difference [39]) in both *E. coli* and *K. pneumoniae*. Closely related strains were detected only in samples from the same WWTP (Additional file 5).

## Discussion

In this study, we describe the phenotypic and genomic characteristics of CP *E. coli* and *K. pneumoniae* isolated from community wastewater influent in ten cities across Finland. We demonstrate the presence of clinically relevant STs and enzyme types of CP *E. coli* and *K. pneumoniae* known to be carried in the population. The results indicate that WWS has the potential to monitor CPE in the population.

In total, 14 different STs of both *E. coli* and *K. pneumoniae* were identified in wastewater. Notably, we detected dominant global STs of CP *E. coli,* such as ST410, ST1284, ST167, and ST405 [15], and CP *K. pneumoniae,* such as ST512, ST15, ST101, and ST307 [14]. Furthermore, we identified several other STs of both *E. coli* and *K. pneumoniae*, some of which have been previously detected in human specimens in Finland (e.g., *E. coli* ST345, ST540, ST617, ST744, and ST1485 and *K. pneumoniae* ST37) (Kati Räisänen, personal communication). The carbapenemases identified in wastewater isolates closely resembled those found in human specimens in Finland, with *bla*_KPC-2_ and *bla*_KPC-3_ being the most prevalent in wastewater. We also detected ST and enzyme type combinations previously linked to clusters in Finland, including *E. coli* ST410 with *bla*_KPC-2_ and *K. pneumoniae* ST512 with *bla*_KPC-3_ [12, 16]. Notably, certain ST and enzyme type combinations found in wastewater, such as *E. coli* ST1284 with *bla*_NDM-5_ in Turku and *K. pneumoniae* ST101 with *bla*_OXA-48_ in Kuopio, have earlier been identified in human specimens from the respective regions (Kati Räisänen, personal communication). Some ST and enzyme type combinations were recurrent in the same WWTP, and some globally dominant STs, *E. coli* ST410 and *K. pneumoniae* ST512 and ST307, were detected in multiple locations. Only some of the recurrent STs in the same WWTP were closely related (< 10 allele difference), whereas in some cases, the isolates belonging to the same ST were phylogenetically distinct. Closely related strains in the same WWTP could originate from one source that is persistently excreting the strain to the wastewater, be a result of persisting strain in the wastewater, or be related to an undetected, potentially local, outbreak in the population. The globally dominant STs between multiple WWTP were genetically distinct, and their occurrence in multiple WWTP could be a consequence of their prevalence in the population. Strains belonging to the epidemiological clusters of *E. coli* ST410 and *K. pneumoniae* ST512 in Finland and an additional *E. coli* ST410 strain distinct from the cluster, were reported in human specimens during the wastewater sampling in 2021–2022 (Kati Räisänen, personal communication). The wastewater strains of *E. coli* ST410 and *K. pneumoniae* ST512 are unlikely linked to at least a single outbreak, as the strains were genetically distinct. However, these strains may represent the diversity of strains circulating in the population. The diversity of strains can be a result of a distinct epidemiological origin of the strains or a natural genetical shift happening in the bacterial population over time [39].

We did not identify known carbapenemases in three *E. coli* and seven *K. pneumoniae* isolates. Resistance mechanisms other than carbapenemase production, such as the loss of outer membrane porins and increased expression of efflux pumps, could contribute to the reduced susceptibility to carbapenems [41]. Furthermore, some isolates may have expressed novel carbapenemases. The limited number of sequenced isolates does not reveal the full diversity of possible STs. Hence, the representation of ST distribution across WWTP may have been biased. Furthermore, strains expressing weaker carbapenemases may be more susceptible to meropenem, and as certain carbapenemases co-occur more commonly with designated STs [15, 42], the selection criteria for sequencing (phenotypical resistance to meropenem) may have influenced the results.

Understanding AMR at the population level and the potential of WWS to act as an early warning tool are the key possibilities of WWS from the public health perspective [23]. Clinical AMR surveillance is crucial for prevention measures in healthcare settings and guiding the treatment of patients. However, clinical surveillance is not particularly suitable for providing an unbiased picture of AMR in the healthy population since the samples are gained from a limited number of individuals who are usually attributed to healthcare and may have a higher probability of carrying AMR bacteria. WWS aims to provide a population-level view with a sample from a larger and more heterogeneous population [23]. Optimally, WWS could assess the incidence and prevalence of CPE in the population and produce descriptive data about the isolates. Moreover, longitudinal and continuous quantitation could reveal the potential trends of CPE occurrence in the population. Here, quantitation was performed with the colony-forming unit (CFU) method, which has limitations, especially when the number of bacteria is low. The method could be further optimized in the future, for example, by accompanying it with molecular methods like qPCR [43]. Describing the STs and enzyme types of the CPE isolates and establishing a phylogenetic comparison scheme (e.g., cgMLST) with CPE isolates from clinical samples and wastewater could reveal the fluctuation of different STs and potential epidemics, especially in long-term surveillance. Quantitative and descriptive WWS could offer valuable early-warning data for healthcare operatives and act as a rationale to increase the clinical surveillance or infection control measures in healthcare settings on a local level. CP *E. coli* and *K. pneumoniae* were present in approximately one-third of the wastewater samples. The prevalence of CPE in the healthy Finnish population is currently unknown, but as CPE is rarely detected in clinical samples, the prevalence is presumably low [12, 44]. Estimating the CPE carriage in the Finnish population with our limited wastewater data is challenging, and the proportion of positive wastewater samples and prevalence in humans in Finland are not directly comparable. For example, some of the detected isolates may have been originally carbapenem-sensitive and have received resistance genes in wastewater through horizontal gene transfer [45]. Furthermore, the dynamics, e.g., the persistence and survival, of clinically relevant AMR bacteria in sewerage systems are not well known, and various factors, such as method sensitivity, unique microbial communities in sewerage systems, and WWTP, as well as conditions in wastewater, such as fluctuating temperature, limited nutrient availability, and chemicals [46], can affect the presence and abundance of CPE in wastewater. These factors impede the assessment of the prevalence of AMR carriage in the community through WWS. The assessment may be less complicated if surveillance is recurrent and long-term, as the identification of trends and epidemiological spikes could be more straightforward [21, 47].

Wastewater is a complex material containing bacteria from various sources [48], and the microbiome can hinder the sensitivity of WWS to detect clinically relevant AMR bacteria. In addition to STs related to humans, we described *E. coli* STs linked both in human and non-human reservoirs (ST137, ST540, ST617, and ST744), animal reservoirs (ST345), environment (ST607), and wastewater systems (ST746 and ST401) [46, 49–51]. Differentiating the bacteria originating from human and non-human sources and further discriminating isolates that have public health relevance is one of the challenges of WWS. Future research should address this challenge by exploring different methods, for example, the potential of utilizing the quantitation of crAssphage, a bacteriophage abundant in the human gut and used in the interpretation of data from WWS of viruses [52]. Furthermore, we identified a diverse array of species in the wastewater, reflecting the complex microbiome of wastewater, which can hamper the identification of the targeted or relevant species. While the culture-based approach provides in-depth knowledge of bacterial STs in wastewater, it requires extensive culturing and utilization of molecular methods (PCR or WGS) to identify all relevant bacterial STs and AMR genes. Continually evolving methodologies, such as culture-enriched metagenomics, HI-C ligation, deep- and long-read sequencing, and single-cell metagenomics, offer potential solutions for these challenges [53–56]. These methodologies, accompanied by artificial intelligence tools, could help to produce population-level data on key infectious agents and their resistance profiles and help to enhance public health security. However, implementing these methodologies requires specialized expertise and extensive resources that may not yet be readily available at the local level, thereby limiting their accessibility. In contrast, the culture-based approach is widely accessible and comparatively low-cost [57], making it a noteworthy option for AMR surveillance, particularly in resource-limited settings.

The selection of approaches and methods in WWS should be guided by specific study objectives, as different approaches provide distinct information about AMR genes, species taxonomy, or bacterial STs and their characteristics. For instance, a gene-based approach may not be optimal for estimating CPE occurrence in the community but could be well-suited for evaluating AMR gene reservoirs or assessing wastewater treatment efficiency. While a culture-based approach alone may not be able to uncover trends in short-term surveillance, it provides information about the epidemiology, abundance, and strain characteristics that could be valuable for health officials or researchers.

## Conclusions

In conclusion, WWS has the potential to monitor CPE in the population. WWS could provide valuable information about the key infectious agents and their resistance profiles at a population level, which offers valuable data from a public health perspective. Furthermore, WWS could identify the geographical hotspots of AMR and guide potential interventions. However, the interpretation of WWS data and the estimations of how well wastewater samples reflect CPE occurrence in the community require further improvements, including enhanced methodologies and optimally also broad screening of CPE in the healthy Finnish population.

### Supplementary Information

**Additional file 1.** Accession numbers for a study deposited in the European Nucleotide Archive (ENA) at EMBL-EBI under accession number PRJEB64775 (https://www.ebi.ac.uk/ena/browser/view/PRJEB64775) and sequencing statistics for whole genome sequenced isolates of *Escherichia coli* (n = 26) and *Klebsiella pneumoniae* (n = 25) from 10 wastewater treatment plants across Finland in 2021–2022.

**Additional file 2.** Additional bacterial species (n = 52) isolated from community wastewater influent samples using ChromAgar mSuperCARBA. Bacterial species were identified using MALDI-TOF MS.

**Additional file 3: A.** Quantity of preliminary* carbapenemase-producing *Escherichia coli* from Espoo, Helsinki, Kuopio, and Lappeenranta wastewater treatment plants in 2021–2022. Nine samplings indicated by mm/yyyy. No visible bar indicates that the quantity was below the detection limit. CFU, colony-forming unit. **B.** Quantity of preliminary* carbapenemase-producing *Escherichia coli* from Oulu,Pietarsaari, and Rovaniemi wastewater treatment plants in 2021–2022. Nine samplings indicated bymm/yyyy. No visible bar indicates that the quantity was below the detection limit. CFU, colony-forming unit. **C.** Quantity of preliminary* carbapenemase-producing *Escherichia coli* from Seinäjoki, Tampere, and Turku wastewater treatment plants in 2021–2022. Nine samplings indicated by mm/yyyy. No visible bar indicates that the quantity was below the detection limit. CFU, colony-forming unit. *Preliminary, as not all isolates were confirmed to carry carbapenemase-encoding genes.

**Additional file 4.** Minimum inhibitory concentration and zone of inhibition of antimicrobials for 50 *Escherichia coli* and 44 *Klebsiella pneumoniae* isolates from 10 wastewater treatment plants across Finland in 2021–2022. Epidemiological cut-off values (ECOFFs) (mg/L and mm) are indicated. ECOFFs in brackets for *K. pneumoniae* differing from ECOFFs for *E. coli.* Isolate ID (identification number) with bold lettering indicates that the isolate was subjected to sequencing. I/D displays insufficient data. COL, Colistin. P/T4, Piperacillin/Tazobactam constant 4. C/T, Ceftolozane/Tazobactam 4. CZA, Ceftazidime/Avibactam. MRP, Meropenem. MRP10 Meropenem (10μg), ERT10 Ertapenem (10μg).

**Additional file 5.** Minimum spanning trees of core genome multilocus sequence typing (cgMLST) of (A) 26 carbapenemase-producing Escherichia coli isolates and (B) 25 carbapenemase-producing Klebsiella pneumoniae isolates. Each circle represents one or multiple identical sequences, and the numbers between the circles indicate the allele differences. Text in the circle indicates the isolate identification number, sample month/year, and city; colors indicate sequence type (ST). A gray background indicates closely related isolates (<10 allele difference). (A) cgMLSTwas based on 2513 columns, pairwise ignoring missing values. (B) cgMLSTwas based on 2365 columns, pairwise ignoring missing values, logarithmic scale.

## Data Availability

The data for this study have been deposited in the European Nucleotide Archive (ENA) at EMBL-EBI under accession number PRJEB64775 (https://www.ebi.ac.uk/ena/browser/view/PRJEB64775). Isolate accession numbers are provided in Additional file 1.

## Abbreviations

AMR: Antimicrobial resistance
BPW: Buffered peptone water
CFU: Colony-forming unit
cgMLST: Core genome multilocus sequence typing
CP: Carbapenemase-producing
CPE: Carbapenemase-producing Enterobacterales
ECDC: European centre for disease prevention and control
KPC: *Klebsiella pneumoniae* carbapanemases
MALDI-TOF MS: Matrix-assisted laser desorption/ionization time-of-flight mass spectrometry
MIC: Minimum inhibitory concentration
MLST: Multilocus sequence typing
NDM: New Delhi metallo-β-lactamases
OXA-48-like: Oxacillinase-48-type carbapenemases
ST: Sequence type
WGS: Whole-genome sequencing
WWS: Wastewater surveillance
WWTP: Wastewater treatment plants
