# Supplementary material for: Clinically relevant sequence types of carbapenemase-producing Escherichia coli and Klebsiella pneumoniae detected in Finnish wastewater in 2021–2022
Source: Antimicrob Resist Infect Control. 2024 Jan 30;13:14. doi: 10.1186/s13756-024-01370-z (PMC10829384; doi:10.1186/s13756-024-01370-z)
Supplement: Supplementary file 3 — Additional file 3: A. Quantity of preliminary* carbapenemase-producing Escherichia coli from Espoo, Helsinki, Kuopio, and Lappeenranta wastewater treatment plants in 2021–2022. Nine samplings indicated by mm/yyyy. No visible bar indicates that the quantity was below the detection limit. CFU, colony-forming unit. B. Quantity of preliminary* carbapenemase-producing Escherichia coli from Oulu,Pietarsaari, and Rovaniemi wastewater treatment plants in 2021–2022. Nine samplings indicated bymm/yyyy. No visible bar indicates that the quantity was below the detection limit. CFU, colony-forming unit. C. Quantity of preliminary* carbapenemase-producing Escherichia coli from Seinäjoki, Tampere, and Turku wastewater treatment plants in 2021–2022. Nine samplings indicated by mm/yyyy. No visible bar indicates that the quantity was below the detection limit. CFU, colony-forming unit. *Preliminary, as not all isolates were confirmed to carry carbapenemase-encoding genes. [file 13756_2024_1370_MOESM3_ESM.pdf]

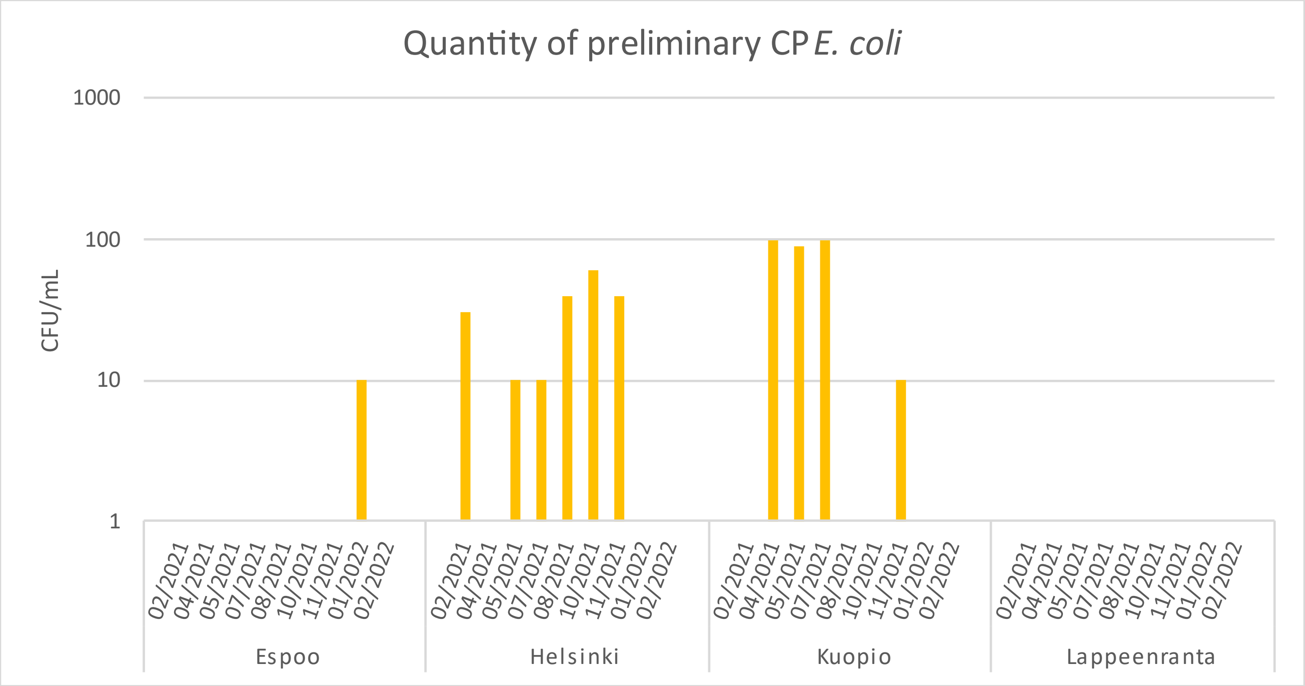

Additional file 3A. Quantity of preliminary carbapenemase-producing Escherichia coli from Espoo, Helsinki, Kuopio, and Lappeenranta wastewater treatment plants in 2021–2022. Nine samplings indicated by mm/yyyy. No visible bar indicates that the quantity was below the detection limit. CFU, colony-forming unit.*

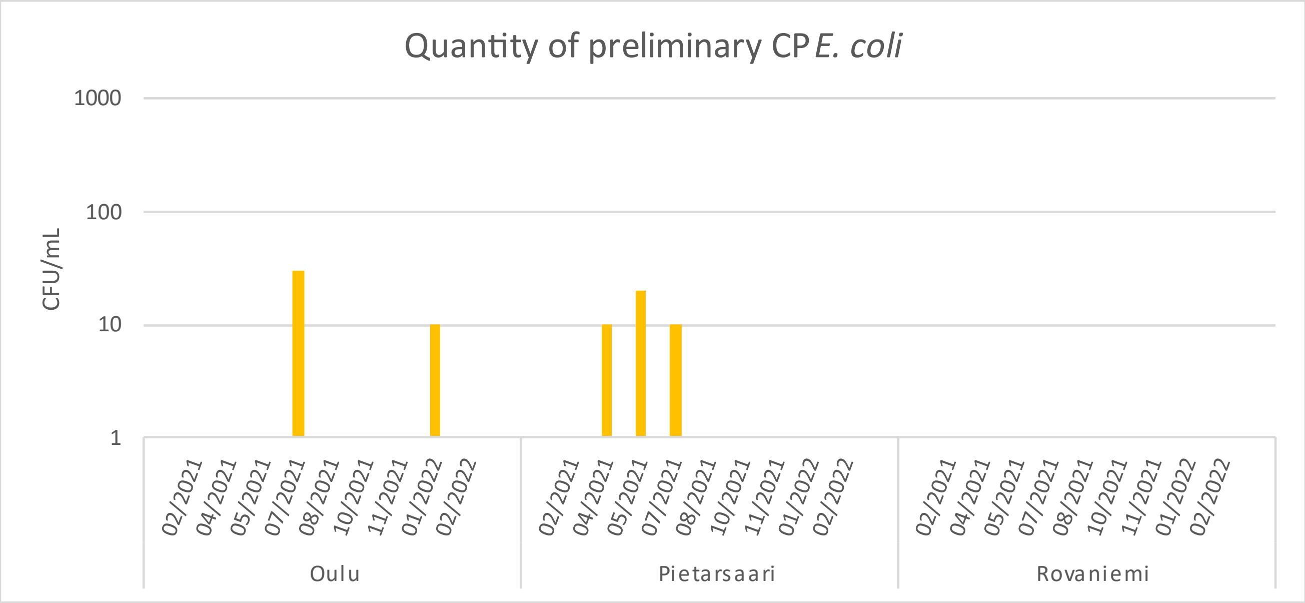

Additional file 3B. Quantity of preliminary carbapenemase-producing Escherichia coli from Oulu, Pietarsaari, and Rovaniemi wastewater treatment plants in 2021–2022. Nine samplings indicated by mm/yyyy. No visible bar indicates that the quantity was below the detection limit. CFU, colony-forming unit.*

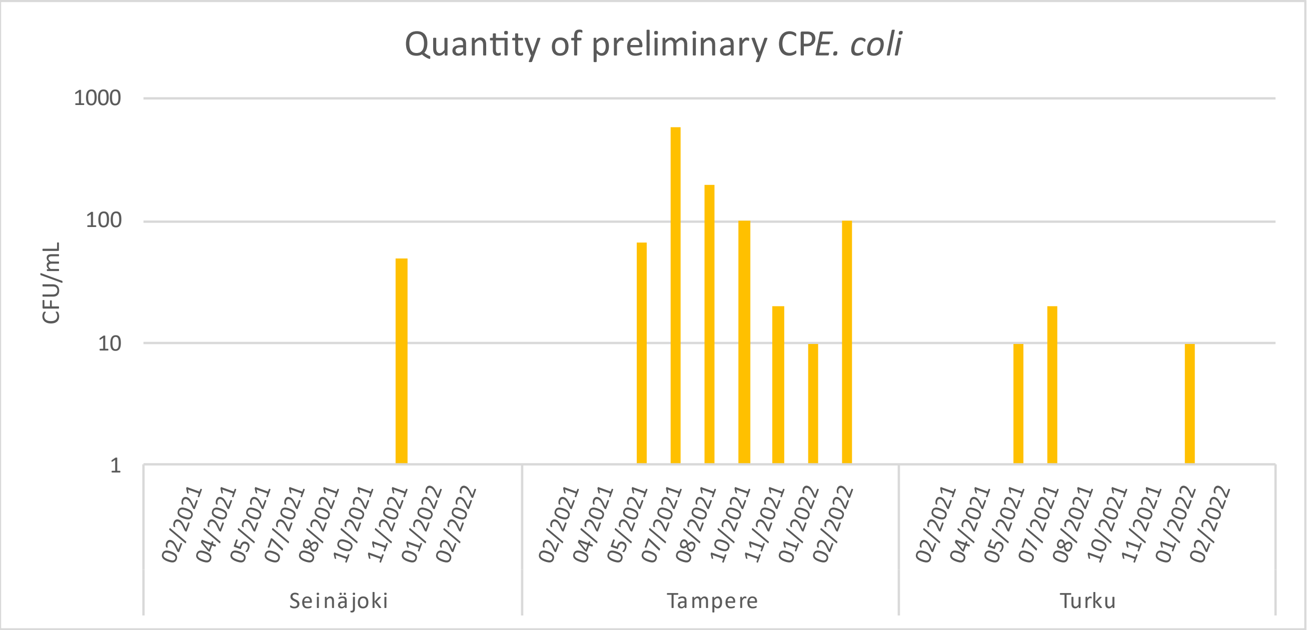

Additional file 3C. Quantity of preliminary carbapenemase-producing Escherichia coli from Seinäjoki, Tampere, and Turku wastewater treatment plants in 2021–2022. Nine samplings indicated by mm/yyyy. No visible bar indicates that the quantity was below the detection limit. CFU, colony-forming unit.*

*Preliminary, as not all isolates were confirmed to carry carbapenemase-encoding genes.
