# Supplementary material for: Clinically relevant sequence types of carbapenemase-producing Escherichia coli and Klebsiella pneumoniae detected in Finnish wastewater in 2021–2022
Source: Antimicrob Resist Infect Control. 2024 Jan 30;13:14. doi: 10.1186/s13756-024-01370-z (PMC10829384; doi:10.1186/s13756-024-01370-z)
Supplement: Supplementary file 4 — Additional file 4. Minimum inhibitory concentration and zone of inhibition of antimicrobials for 50 Escherichia coli and 44 Klebsiella pneumoniae isolates from 10 wastewater treatment plants across Finland in 2021–2022. Epidemiological cut-off values (ECOFFs) (mg/L and mm) are indicated. ECOFFs in brackets for K. pneumoniae differing from ECOFFs for E. coli. Isolate ID (identification number) with bold lettering indicates that the isolate was subjected to sequencing. I/D displays insufficient data. COL, Colistin. P/T4, Piperacillin/Tazobactam constant 4. C/T, Ceftolozane/Tazobactam 4. CZA, Ceftazidime/Avibactam. MRP, Meropenem. MRP10 Meropenem (10μg), ERT10 Ertapenem (10μg). [file 13756_2024_1370_MOESM4_ESM.pdf]

Additional file 4. Minimum inhibitory concentration and zone of inhibition of antimicrobials for 50 Escherichia coli and 44 Klebsiella pneumoniae isolates from 10 wastewater treatment plants across Finland in 2021-2022.

Isolate ID	City	Time (mm/yy)	Species	MIC (mg/L)					Zone of inhibition (mm)	
				COL	P/T4	C/T	CZA	MRP	MRP10	ERP10
(ECOFFs)				2	8	1	ID	0.06 (0.125)	26 (24)	23 (21)
E1	Helsinki	02/21	*E. coli*	8	> 32	> 8	2	1	21.7	17.2
**E2**	Helsinki	02/21	*E. coli*	0.5	> 32	> 8	8	2	25.3	18.2
**E3**	Kuopio	04/21	*E. coli*	≤ 0.25	32	> 8	> 16	≤ 0.12	19.9	14.8
**E4**	Pietarsaari	04/21	*E. coli*	≤ 0.25	> 32	> 8	> 16	> 16	10.9	6.5
**E5**	Helsinki	05/21	*E. coli*	0.5	> 32	8	≤ 1	1	20	16
**E6**	Kuopio	05/21	*E. coli*	≤ 0.25	32	> 8	> 16	≤ 0.12	20	15
E7	Pietarsaari	05/21	*E. coli*	≤ 0.25	> 32	> 8	> 16	1	16	13
**E8**	Pietarsaari	05/21	*E. coli*	≤ 0.25	> 32	> 8	> 16	16	12	6
E9	Tampere	05/21	*E. coli*	0.5	> 32	> 8	≤ 1	1	20	16
E10	Tampere	05/21	*E. coli*	0.5	> 32	> 8	≤ 1	1	20	16
**E11**	Tampere	05/21	*E. coli*	0.5	> 32	> 8	≤ 1	1	19	16
**E12**	Turku	05/21	*E. coli*	≤ 0.25	> 32	> 8	> 16	8	15	6
**E13**	Helsinki	07/21	*E. coli*	4	> 32	> 8	4	4	22.2	14.8
**E14**	Oulu	07/21	*E. coli*	≤ 0.25	> 32	> 8	≤ 1	0.5	23.7	18
**E15**	Pietarsaari	07/21	*E. coli*	0.5	> 32	> 8	> 16	> 16	12	6
E16	Tampere	07/21	*E. coli*	0.5	> 32	> 8	≤ 1	0.5	21.1	19.6
**E17**	Tampere	07/21	*E. coli*	0.5	> 32	> 8	≤ 1	2	18.7	17
E18	Tampere	07/21	*E. coli*	0.5	> 32	> 8	≤ 1	1	19	16.8
**E19**	Turku	07/21	*E. coli*	0.5	> 32	2	≤ 1	0.25	24.2	19.7
E20	Helsinki	08/21	*E. coli*	4	> 32	> 8	4	4	24.4	15.7
**E21**	Helsinki	08/21	*E. coli*	≤ 0.25	> 32	> 8	4	8	17.4	7.3
E22	Tampere	08/21	*E. coli*	0.5	> 32	> 8	≤ 1	2	25.1	18.7
**E23**	Tampere	08/21	*E. coli*	1	> 32	> 8	≤ 1	8	21.8	13.3

E24	Tampere	08/21	*E. coli*	1	4	0.5	≤ 1	≤ 0.12	41.1	38
E25	Tampere	08/21	*E. coli*	0.5	> 32	> 8	≤ 1	4	25.9	18.4
**E26**	Helsinki	10/21	*E. coli*	2	> 32	> 8	4	4	26.4	18.2
E27	Helsinki	10/21	*E. coli*	0.5	> 32	> 8	8	2	28.3	16.7
E28	Helsinki	10/21	*E. coli*	8	> 32	> 8	8	2	26.8	17
E29	Helsinki	10/21	*E. coli*	0.5	> 32	> 8	≤ 1	≤ 0.12	35	25.2
E30	Tampere	10/21	*E. coli*	0.5	> 32	> 8	8	2	21.2	17.2
E31	Tampere	10/21	*E. coli*	0.5	8	0.5	≤ 1	≤ 0.12	36.7	34.8
E32	Tampere	10/21	*E. coli*	1	> 32	> 8	≤ 1	2	21.5	17.5
**E33**	Tampere	10/21	*E. coli*	0.5	> 32	> 8	≤ 1	4	21.6	18.7
E34	Tampere	10/21	*E. coli*	0.5	> 32	> 8	≤ 1	1	26.3	22.3
**E35**	Helsinki	11/21	*E. coli*	≤ 0.25	> 32	> 8	4	2	21.5	14.6
E36	Helsinki	11/21	*E. coli*	2	> 32	0.5	≤ 1	0.5	22.7	20.2
**E37**	Kuopio	11/21	*E. coli*	≤ 0.25	> 32	> 8	> 16	16	17.2	14.3
E38	Seinäjoki	11/21	*E. coli*	≤ 0.25	> 32	0.5	≤ 1	0.5	28.8	25.1
E39	Seinäjoki	11/21	*E. coli*	0.5	> 32	0.5	≤ 1	0.25	26.7	26.1
E40	Seinäjoki	11/21	*E. coli*	≤ 0.25	> 32	0.5	≤ 1	0.25	27.3	25.6
**E41**	Seinäjoki	11/21	*E. coli*	≤ 0.25	> 32	0.5	≤ 1	0.5	27.8	25.3
E42	Seinäjoki	11/21	*E. coli*	0.5	> 32	0.5	≤ 1	0.5	28.2	26.8
**E43**	Tampere	11/21	*E. coli*	0.5	> 32	> 8	≤ 1	2	14.7	13.6
E44	Tampere	11/21	*E. coli*	0.5	> 32	> 8	8	1	15.6	16.2
**E45**	Espoo	01/22	*E. coli*	0.5	4	0.5	≤ 1	≤ 0.12	29.2	26.7
**E46**	Oulu	01/22	*E. coli*	0.5	> 32	> 8	> 16	> 16	14.3	12.9
**E47**	Tampere	01/22	*E. coli*	0.5	> 32	> 8	≤ 1	4	16.9	13.8
**E48**	Turku	01/22	*E. coli*	0.5	> 32	> 8	> 16	> 16	15	9.9
E49	Tampere	02/22	*E. coli*	0.5	> 32	> 8	8	1	21.6	15.5
**E50**	Tampere	02/22	*E. coli*	0.5	> 32	> 8	8	1	21	16.5
**K1**	Rovaniemi	02/21	*K. pneumoniae*	> 8	> 32	> 8	4	16	12.9	9.63
**K2**	Lappeenranta	04/21	*K. pneumoniae*	0.5	32	2	≤ 1	0.5	26.0	19.3
**K3**	Rovaniemi	04/21	*K. pneumoniae*	> 8	> 32	> 8	4	8	14.8	9.5

**K4**	Turku	04/21	*K. pneumoniae*	0.5	> 32	1	2	1	24.4	15.9
**K5**	Espoo	05/21	*K. pneumoniae*	> 8	> 32	> 8	2	> 16	9	6
K6	Espoo	05/21	*K. pneumoniae*	> 8	> 32	> 8	2	> 16	9	6
K7	Espoo	05/21	*K. pneumoniae*	> 8	> 32	> 8	2	> 16	9	6
K8	Espoo	05/21	*K. pneumoniae*	> 8	> 32	> 8	2	> 16	9	6
**K9**	Kuopio	05/21	*K. pneumoniae*	> 8	> 32	> 8	≤ 1	> 16	10	6
K10	Rovaniemi	05/21	*K. pneumoniae*	≤ 0.25	> 32	> 8	≤ 1	4	18	16
**K11**	Rovaniemi	05/21	*K. pneumoniae*	≤ 0.25	> 32	> 8	4	4	16	13
K12	Rovaniemi	05/21	*K. pneumoniae*	≤ 0.25	> 32	> 8	≤ 1	4	18	16
K13	Rovaniemi	05/21	*K. pneumoniae*	> 8	> 32	> 8	8	4	16	13
**K14**	Turku	05/21	*K. pneumoniae*	0.5	> 32	> 8	≤ 1	4	18	15
**K15**	Helsinki	07/21	*K. pneumoniae*	0.5	> 32	2	≤ 1	2	18.3	6
K16	Rovaniemi	07/21	*K. pneumoniae*	0.5	> 32	> 8	≤ 1	2	19.9	19
K17	Rovaniemi	07/21	*K. pneumoniae*	0.5	> 32	> 8	≤ 1	2	21.9	21.8
K18	Rovaniemi	07/21	*K. pneumoniae*	0.5	> 32	> 8	≤ 1	2	16.6	16.9
**K19**	Rovaniemi	07/21	*K. pneumoniae*	0.5	> 32	> 8	4	4	14.7	13.3
**K20**	Tampere	07/21	*K. pneumoniae*	0.5	> 32	> 8	≤ 1	1	22.7	17.9
**K21**	Oulu	08/21	*K. pneumoniae*	0.5	> 32	> 8	4	16	9	6
K22	Oulu	08/21	*K. pneumoniae*	0.5	> 32	> 8	≤ 1	8	19.5	13.4
**K23**	Tampere	08/21	*K. pneumoniae*	2	> 32	> 8	≤ 1	4	18.7	11.1
K24	Tampere	08/21	*K. pneumoniae*	≤ 0.25	8	0.5	≤ 1	≤ 0.12	34.7	23.6
**K25**	Turku	08/21	*K. pneumoniae*	0.5	16	2	≤ 1	> 16	11.8	6
**K26**	Helsinki	10/21	*K. pneumoniae*	2	> 32	> 8	≤ 1	8	24	11.2
**K27**	Lappeenranta	10/21	*K. pneumoniae*	0.5	16	4	≤ 1	1	24.2	11.7
**K28**	Rovaniemi	10/21	*K. pneumoniae*	> 8	> 32	> 8	4	8	19.5	13.4
K29	Rovaniemi	10/21	*K. pneumoniae*	0.5	> 32	> 8	≤ 1	4	20.5	16.9
K30	Rovaniemi	10/21	*K. pneumoniae*	0.5	> 32	> 8	≤ 1	4	23	17.7
**K31**	Turku	10/21	*K. pneumoniae*	0.5	> 32	> 8	≤ 1	8	18.3	15
**K32**	Helsinki	11/21	*K. pneumoniae*	1	> 32	2	≤ 1	2	22.7	18.7
**K33**	Oulu	11/21	*K. pneumoniae*	≤ 0.25	> 32	> 8	8	> 16	6	6

K34	Rovaniemi	11/21	*K. pneumoniae*	0.5	> 32	> 8	≤ 1	4	16.8	16.1
**K35**	Rovaniemi	11/21	*K. pneumoniae*	0.5	> 32	> 8	4	16	15	13.8
K36	Rovaniemi	11/21	*K. pneumoniae*	0.5	> 32	> 8	≤ 1	2	14.5	16
K37	Rovaniemi	11/21	*K. pneumoniae*	0.5	> 32	> 8	≤ 1	4	16.3	15.4
K38	Helsinki	01/22	*K. pneumoniae*	0.5	> 32	2	≤ 1	1	20.7	17.5
**K39**	Helsinki	01/22	*K. pneumoniae*	0.5	> 32	2	≤ 1	4	15.6	10.6
K40	Helsinki	01/22	*K. pneumoniae*	0.5	> 32	2	≤ 1	2	21.3	16.2
K41	Rovaniemi	01/22	*K. pneumoniae*	> 8	> 32	> 8	8	8	12.4	11
**K42**	Rovaniemi	01/22	*K. pneumoniae*	> 8	> 32	> 8	4	16	10.9	7.9
**K43**	Espoo	02/22	*K. pneumoniae*	0.5	> 32	> 8	8	16	9.2	7.9
**K44**	Tampere	02/22	*K. pneumoniae*	0.5	> 32	> 8	1	2	14.2	14

Epidemiological cut-off values (ECOFFs) (mg/L and mm) are indicated. ECOFFs in brackets for K. pneumoniae differing from ECOFFs for E. coli. Isolate ID (identification number) with bold lettering indicates that the isolate was subjected to sequencing. I/D displays insufficient data. COL, Colistin. P/T4, Piperacillin/Tazobactam constant 4. C/T, Ceftolozane/Tazobactam 4. CZA, Ceftazidime/Avibactam. MRP, Meropenem. MRP10 Meropenem (10µg), ERT10 Ertapenem (10µg).
