# Supplementary figures and images for: Clinically relevant sequence types of carbapenemase-producing Escherichia coli and Klebsiella pneumoniae detected in Finnish wastewater in 2021–2022
Source: Antimicrob Resist Infect Control. 2024 Jan 30;13:14. doi: 10.1186/s13756-024-01370-z (PMC10829384; doi:10.1186/s13756-024-01370-z)

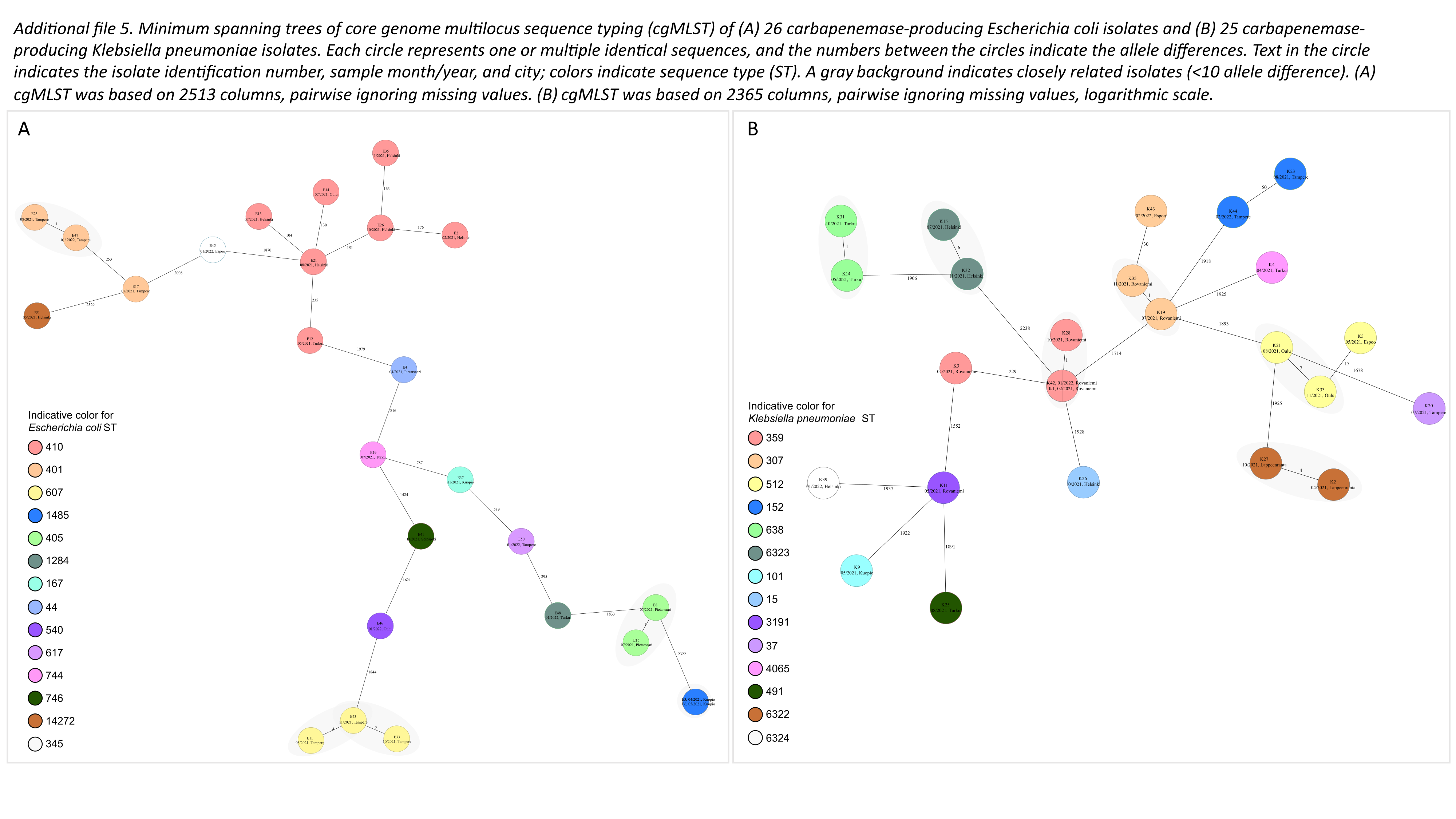

Supplement: Supplementary file 5 — Additional file 5. Minimum spanning trees of core genome multilocus sequence typing (cgMLST) of (A) 26 carbapenemase-producing Escherichia coli isolates and (B) 25 carbapenemase-producing Klebsiella pneumoniae isolates. Each circle represents one or multiple identical sequences, and the numbers between the circles indicate the allele differences. Text in the circle indicates the isolate identification number, sample month/year, and city; colors indicate sequence type (ST). A gray background indicates closely related isolates (<10 allele difference). (A) cgMLSTwas based on 2513 columns, pairwise ignoring missing values. (B) cgMLSTwas based on 2365 columns, pairwise ignoring missing values, logarithmic scale. [file 13756_2024_1370_MOESM5_ESM.png]
